# EHD instability of a cylindrical interface separating two couple-stress fluids

**DOI:** 10.1038/s41598-024-56143-w

**Published:** 2024-03-07

**Authors:** Galal M. Moatimid, Mohamed F. E. Amer, Doaa A. Ibrahim

**Affiliations:** 1 Department of Mathematics, Faculty of Education, Ain Shams University, Roxy, Cairo, Egypt; 2 Department of Mathematics and Computer Science, Faculty of Sciences, Beni-Suef University, Beni-Suef, 62511 Egypt

**Keywords:** Electrohydrodynamics, Hydrodynamic instability, Couple-stress fluids, Viscous potential theory, Porous media, Physics, Fluid dynamics, Applied mathematics

## Abstract

This article is an attempt at examining the axi-symmetric and asymmetric streaming flows described by the CSF framework. A liquid that has microfibers implanted in it, like a fiber-reinforced composite substance, is so-called CSF. It is a system that consists of an endless vertical cylindrical interface that separates the two CSF structure. The CSFs are increasingly growing significant in modern manufacturing and technology, necessitating greater research into these fluids. An axial EF acts over the cylindrical contact in addition to the influence of CSF. The VPT is employed for the sake of convenience to minimize mathematical complexity. Combining the elementary linear equations of motion and the proper linear related BCs is the major procedure of the linear technique. A collection of physically dimensionless numbers is produced using a non-dimensional process. Subsequently, the requirements for hypothetical linear stability are developed. With the aid of the Gaster's theorem, the MS is applied in computing the dispersion relationships. After carefully examining a variety of effects on the stability investigation of the system at issue, it has been shown that the system is more unstable when a porous material is present than it would be without one. The resulting axisymmetric disturbance situation is more unstable. The linear techniques are depicted throughout a number of graphs.

## Introduction

A large field of research known as fluid mechanics examines fluids (liquids and gases), whether they are at rest or moving. It can be used in many different domains, including biological engineering, mechanical, chemical, agricultural, and food science engineering, as well as aerodynamics and bio-fluid mechanics. The Navier–Stokes equations were the basic equations of motion of the Newtonian category. There were no precise answers to these equations. Only a small number of limited issues have universal solutions. As was common knowledge, the precise solutions hold great importance. Not only do they symbolize the fundamental flow phenomena, but they can also serve as evidence for solutions derived from a variety of methodologies. It gets more challenging to find the exact solutions in non-Newtonian flow circumstances. The relationship involving stress and strain in the latter fluids is nonlinear. Couple stress fluids (CSFs), which originally appeared^1^, have attracted a lot of attention due to their various properties, including body couples, non-symmetric stress tensors, and couple stresses. Because of its many industrial and scientific uses, including the extraction of polymer fluids, the solidification of liquid crystals, and the extraction of animal blood, CSFs have drawn attention. A few precise CSF solutions were discovered^2^. Investigators looked at how a chemical reaction and an external vertical magnetic field (MF) affected the CSFs between infinite horizontal parallel plates to cause double-diffusive convection to begin^3^. An inclined plane's ability to support a thin non-Newtonian liquid layer flow was examined^4^. The evolution differential equation regulating the behavior of a thin film of a CSF, which provided the time record of the interface characteristics, was found within the structure of the long wave approximations. A numerical investigation was conducted to examine the impact of a consistent vertical magnetic field (MF) on the stability of pressure-driven non-Newtonian fluid flow in an isothermal conduit that conducts electricity^5^. The CSF theory, which allows for polar effects and is frequently seen in liquids containing very big molecules, was used to represent the non-Newtonian fluid. It investigated how the CSF flowed while taking into account varying viscosity and a uniform axial EF^6^. It was found that raising the coupling stress parameter and the viscosity fluctuation parameter improved the velocity, temperature, and overall rate of heat transfer across the channel. It was decided to use the linear/non-linear stability analysis technique on a CSF layer whose viscosity varied with temperature and pressure^7^. It was discovered that the linear and nonlinear thresholds that capture the mechanics of the convection initiation are identical. It examined how the CSFs affected the magnetized ferrofluid's convective stability for various bounding surface configurations^8^. To identify eigenvalue problems, both linear and nonlinear analyses were performed. In general, the study of CSFs has significance for engineering, biology, materials science, and other fields by providing an understanding of the extensive and complicated behavior of non-Newtonian fluids in a variety of physical environments.

Electrohydrodynamics (EHD), as a branch of fluid mechanics, examines the effects of EF on fluids. The EHD was a combination of these two sciences since many attractive problems in it require both the action of the EF and the movement of fluids. EHD incorporates the complex relationship between internal, viscosity, and electric power. It produced visually striking phenomena in equipment used in drop-sparing and inkjet printing. The EHD thermal instability in a horizontal layer of an elastic viscous nanofluid saturating a porous medium was investigated under the effect of a vertical AC EF^9^. A Darcy model has been applied to a porous media, and a CSF model was used to characterize the rheological behavior of nanofluid. Researchers examined the convection instability in an EF-modulated horizontal dielectric CSF layer^10^. It was demonstrated that by appropriately adjusting several control parameters, the beginning of convection can be delayed or expedited. A mathematical model was used to study the combined effects of the axial EF and the transverse MF on two-dimensional micro-peristaltic channels with different peristaltic wave propagations at the left and right channel walls of a CSF^11^. To see if it had entered the system, a constant axial EF was established^12^. Their approach produced a large number of non-dimensional quantities. In recognition of this work, scientists now have a good understanding of how viscous fluids move in cylinders and turn unstable when subjected to EFs. Examiners investigated the stability of a perfect gas in the upper layer and a viscous fluid at the bottom of two horizontal fluids superposed one above the other^13^. Coriolis and centrifugal forces were considered. Another typical, normal EF that affected the system was this one. In EHD instability, a vertical cylindrical interface was discussed^14^. An even axial EF had an impact on the system that was being examined. There was a combination of the traditional normal modes analysis and the implication of the viscous potential theory (VPT). This methodology is used in the current inquiry because of how important the EF's presence is. A statistical and analytical study was conducted on the EHD of thermal stability of a viscoelastic nanofluid overflowing a porous with vertical AC of EF^15^. For porous media, the Brinkman type was used, and the CSF model was used to explain the rheological behavior of the nanofluid. Both theoretical and quantitative study was done on the viscoelastic liquid film of the CSF type moving with relative motion through a permeable media into a perfect gas^16^. Analytical solutions for both axisymmetric and asymmetric disturbances were found. Unlike the previous work, the current problem incorporates the energy equation to find the temperature distribution because of the importance of heat transmission in many real applications. It was discussed how vertical cylindrical EHD instability occurred^14^. A regular axial EF had an impact on the system that was being studied. The thermo-capillary phenomenon was produced by incorporating the effect of heat transfer into the buoyancy term and the ST parameter. The fundamental equations included an energy equation since temperature transfer has so many practical uses. It investigated how a rotating ring of double micro-layers of gas and fluid may maintain temporal stability^17–19^.

Numerous stability challenges have been looked into using the VPT. The VPT generated the Navier–Stokes equation solution for liquids with precisely zero vortices. The VPT solely considered normal stresses; balancing and tangential stresses were not considered. The RTI of two viscous fluids was examined using VPT^20^. The Rayleigh–Taylor instability (RTI) of the viscoelastic fluid issue was expanded^21^, and it was revealed that the viscoelastic potential theory offers the critical wavelength as well as a growth level that is within 10% of the correct theory. The solution to this issue was excellently examined^22^. The VPT was used to model the stability of slim, viscid, and dielectric fluid sheets^23^. The structural investigation revealed that although liquid viscosity stabilizes structures, air viscosity destabilizes them. A study on the linear EHD stability of a boundary between two viscid films looked at the VPT^24^. The impacts of various settings on stability were displayed in a series of graphs. When compared to a typical Newtonian viscous fluid, it was realized that the combination of the EF and couple stress was more powerful at stabilizing the weakly conducting CSF. It was discovered that the separate kinetic energy spectrum components were examined and displayed for various parametric values to obtain comprehensive data at the fluid flow critical condition^25^. It was thought to be a film of CSF heated from the bottom in porous media^26^. A worldwide nonlinear stability study of a CSF layer penetrating a permeable medium with viscosity that depends on temperature and pressure was performed^27^. For all the various conducting boundary systems, it was discovered that the Darcy-Brinkman model produced a system that was thermally more reliable than the Darcy prototype. How the MF affected the thermal convection of a CSF saturating a porous media was examined^28^. Because of the significance of porous relationships, this methodology will be used in the present study. A permeable stretched sheet in motion inside a porous medium caused a non-Newtonian Maxwell fluid to flow was investigated^29^. The realization of an increased heat transfer rate has become a major problem in the domain of thermal technological advances, which have faced numerous challenges in recent decades. Often, heating conventional fuels produces temperatures that are too high for renewable energy to reach^30^. An asymmetric channel's non-Newtonian nanofluid behavior brought on by peristaltic waves was examined^31^. The generation of heat radiation and activation energy was also taken into account. The electro-osmotic flow of immiscible fluids across a porous material in vertical annular microtubes was visualized using a numerical simulation^32^.

As aforementioned, CSF has several industrial uses. Given the importance and uses of electrified CSFs and flows through porous media in advanced technologies, the purpose of this study is to examine the EHD stability of two cylindrical dielectric CSFs flowing over permeable media. To the best of our knowledge, despite the study's relevance for biological fluid flows, there is no literature on the simultaneous presence of CSFs and EF on the pressure-driven stability of fluid flow in a cylindrical channel. This paper's innovation is in demonstrating the VPT's validity while examining the CSFs' stability features. The main objective of the present study is to urge readers to seek suitable answers to the following queries:

- What standards does the linear instability method use?
- How many physically non-dimensional numerals are there in the linear approach?
- How does the concept of linear stability work?

The article is structured as follows: In “Construction of the problem”, details regarding the physical system and mathematical model are supplied, in addition to base state profiles and linearized equations for the disturbed state. In “BCs and dispersion relation”, a linear specific equation of the interface displacement is obtained using the boundary conditions (BCs) which are also provided. The transcendental dispersion relation is also provided. In “Discussions of outcomes”, the linear stability approach is developed, and the findings are graphically displayed to demonstrate the impact of unlike factors on the instability profile. In “Concluding remarks”, concluding remarks and key findings are included.

## Construction of the problem

**Figure 1:**
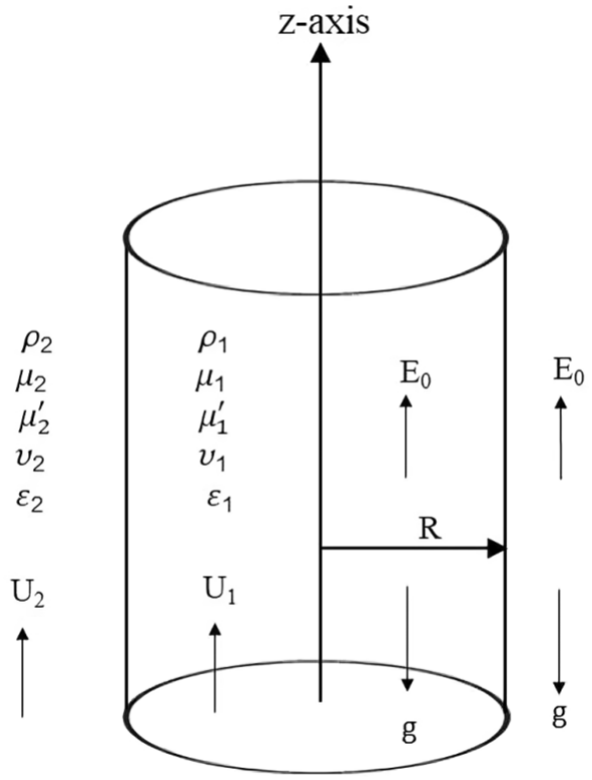
Model shown in their unaltered state.

The current study examines a system involving two types of fluids: liquid and gas, called streaming CSFs. These fluids flow uniformly in parallel along a jet's axis. The inner (liquid) and outer (gas) fluids have different characteristics. Therefore, the study addresses itself to a system with two homogeneous, incompressible, dielectric, and streaming CSFs uniformly moving parallel to the axis of the jet. The parameters in the inner (Liquid) and outer (Gas) fluids are denoted by the subscripts 1 and 2, respectively. Throughout the following formulation, $\mu$ and $\mu^{\prime}$ are typically the dynamic viscosity and viscoelasticity of CSF viscous terms, and $\underline{v}$ is the velocity of CSF Darcian velocity of the liquid motion. In porous media, where permeability is indicated by the symbol $\lambda$, the flows are saturated. Porosity can be treated as a unit. The two cylinder-shaped fluids flow at uniform velocities $U_{1}$ and $U_{2}$. Density and dielectric constants are referred to as $\rho \,$ and $\varepsilon$, respectively. A consistent axial EF $E_{0}$ is parallel to the interface between the two media. The gravity force $g$ affecting the opposite $z -$ direction is considered. To study the system, cylindrical polar coordinates are used for their practicality. The axis aligns with the axis of symmetry, simplifying the analysis of flow dynamics and interactions. The axis $z -$ is drawn parallel to the axis of symmetry. Figure 1 provides a drawing of the working prototype.

The common stability methodology motivation is created utilizing a consistent approach that acknowledges Chandrasekhar's groundbreaking work^33^. In light of this, any involved function might be written as:

1 $$f(r,\,\theta ,\,z;\,\,t) = F(r)\exp \,\left[ {i(k\,z + n\theta ) + \omega t} \right] + c.c.,$$

where $f$ denotes any physical quantity.

In the assessment of the temporal controlling disturbance, the wave numeral is regarded as a positive real value. Both $k$ and $\omega$ are anticipated to be complex as: $\omega = \omega_{r} + i\omega_{i}$ and $k = k_{r} + ik_{i}$ through the review of spatial–temporal instability. The spatial growth of instability in addition to the stream direction is therefore produced by $k_{i} > 0$, the time growth of instability by $\omega_{r} > 0$, and the two together are produced by Refs.^34,35^.

The CSF motion is governed by the following equations^36^:

2 $$\rho_{j} \left[ {\frac{{\partial \underline{{\tilde{v}}}_{j} }}{\partial t} + (\underline{{\tilde{v}}}_{j} .\nabla )\underline{{\tilde{v}}}_{j} } \right] = - \nabla \tilde{p}_{j} + \rho_{j} \underline{g} - \frac{1}{\lambda }\,\,\left( {\mu_{j} - \mu^{\prime}_{j} \,\nabla^{2} } \right)\underline{{\tilde{v}}}_{j} ,\,\,j = 1,\,\,{\text{and}}\,\,2\,\,$$

The equation of continuity exists in^37^

3 $$\nabla .\underline{{\tilde{v}}}_{j} = 0\,,\,\,\,\,\,\,\,$$

in which $\underline{{\tilde{v}}}_{j}$ is the fluid velocities, $\tilde{p}_{j}$ is the pressures, and $\underline{{\text{g}}} = (0,0, - g)$ is the acceleration of gravity.

In formulating the basic governing equation of motion as given in Eq. (2), the VPT is utilized.

Euler equations are derived from the Navier–Stokes equations by employing the VPT, as previously shown^38–40^. Consequently, the viscous fluid stress tensor in the momentum equation is eliminated in the main formulation of the foundational equation of the flow. Therefore, the basic equation is the formula that, while taking into consideration the Brinkman-Darcy rule, determines how well a viscous incompressible liquid flows across porous media. Consequently, the fluids are thought to be irrotational under the principles of the VPT. When a cylindrical interface is found at the two undisturbed cylindrical interfaces, we assume that the three liquid phases are immiscible and undisturbed. A similar process was effectively applied to move from perturbation theory to the viscoelastic fluid^41^. Given that it can be reasonably presumed that the movements elsewhere are irrotational**, t**his approach was predicated by Batchelor's concept of VPT^42^. In this problem, the derivations are fully suitable to the VPT, without requiring the intricate adjustment of the boundary layer formulas for the low upward flow. Since the equations controlling the irrotational flow generate the Laplace formula, it should be feasible to alter the BCs at the interface including minor viscoelastic effects. Consequently, the viscoelastic influences can be formulated using the normal stress BC from the current work. Assuming the viscoelastic concept, the primary equations about the common fluid phases were supplied.

Because of the apparent EF intensity in this circumstance, the reported Maxwell's formulas must be involved. Melcher^43^ published an innovative book that examined the surface waves of MHD and EHD in depth. Here, the influence of the EF is currently used. As a result, the MF effect may be discounted. Maxwell equations are simplified as follows:

4 $$\nabla .(\varepsilon_{j} \,\underline{E}_{j} ) = 0\quad \& \quad \nabla \times \underline{E}_{j} = 0$$

Consequently, $\underline{E}_{j}$ the EF might be expressed concerning a scalar function $\psi_{j}$ as

5 $$\underline{E}_{j} = E_{0\,} \underline{e}_{z} - \nabla \tilde{\psi }_{j} .$$

Equation (4) implies that the EF's potential complies with Laplace's equation.

6 $$\nabla^{2} \,\tilde{\psi }_{j} = 0$$

Let the perturbation equations be as follows to examine the above system of equations:

7 $$\underline{{\tilde{v}}}_{j} = (0,0,U_{j} ) + \underline{v}_{j} \quad {\text{where}}\quad \underline{v}_{j} = \underline{V}_{j} (r)\,Exp\,\left[ {i(kz + n\theta ) + \omega t} \right]$$

8 $$\tilde{p}_{j} \, = P_{0j} + \,p_{j} \quad {\text{where}}\quad p_{j} = P_{j} (r)\,Exp\,\left[ {i(kz + n\theta ) + \omega t} \right]$$

9 $${\text{and}}\quad \tilde{\psi }_{j} = \Psi_{0j} + \psi_{j} \quad {\text{where}}\quad \psi_{j} = \Psi_{j} (r)\,Exp\,\left[ {i(kz + n\theta ) + \omega t} \right]$$

In light of the cylindrical coordinates, the continuity and momentum equations are provided by expressing the velocity in a proper component form in Eqs. (2) and (3).

10 $$\frac{{\partial \,v_{rj} }}{\partial r} + \frac{{v_{rj} }}{r} + \frac{1}{r}\frac{{\partial v_{\theta j} }}{\partial \theta } + \frac{{\partial \,v_{zj} }}{\partial z} = 0,$$

11 $$\rho_{j} \left( {\frac{\partial \,}{{\partial t}} + U_{j} \frac{\partial \,}{{\partial \,z}}} \right)v_{rj} = - \frac{{\partial \,p_{j} }}{\partial \,r} - \frac{{\mu_{j} }}{\lambda }v_{rj} + \frac{{\mu^{\prime}_{j} }}{\lambda }\left[ {\nabla^{2} v_{rj} - \frac{{v_{rj} }}{{r^{2} }} + - \frac{2}{{r^{2} }}\frac{{\partial v_{\theta j} }}{\partial \theta }} \right],$$

12 $$\rho_{j} \left( {\frac{\partial \,}{{\partial t}} + \,\,U_{j} \frac{\partial \,}{{\partial \,z}}} \right)v_{\theta j} = - \frac{1}{r}\frac{{\partial \,p_{j} }}{\partial \,\theta } - \frac{{\mu_{j} }}{\lambda }v_{\theta j} + \frac{{\mu^{\prime}_{j} }}{\lambda }\left[ {\nabla^{2} v_{\theta j} - \frac{{v_{\theta j} }}{{r^{2} }} + + \frac{2}{{r^{2} }}\frac{{\partial v_{rj} }}{\partial \theta }} \right],$$

13 $$\rho_{j} \left( {\frac{\partial \,}{{\partial t}} + U_{j} \frac{\partial \,}{{\partial \,z}}} \right)v_{zj} = - \frac{{\partial \,p_{j} }}{\partial \,z} - \frac{{\mu_{j} }}{\lambda }v_{zj} + \frac{{\mu^{\prime}_{j} }}{\lambda }\nabla^{2} v_{zj} ,$$

In which $$\nabla^{2} = \frac{{\partial^{2} }}{{\partial r^{2} }} + \frac{1}{r}\frac{\partial }{\partial r} + \frac{1}{{r^{2} }}\frac{{\partial^{2} }}{{\partial \theta^{2} }} + \frac{{\partial^{2} }}{{\partial z^{2} }}$$.

Take the divergence of both sides of Eq. (2) and use Eq. (8) to solve the preceding system of equations.

14 $$\nabla^{2} p_{j} = 0,$$

15 $${\text{and}}\quad \frac{1}{r}\left[ {\frac{\partial }{\partial r}\left( {r\frac{{\partial p_{j} }}{\partial r}} \right) + \frac{1}{r}\frac{{\partial^{2} p_{j} }}{{\partial \theta^{2} }} + r\frac{{\partial^{2} p_{j} }}{{\partial z^{2} }}} \right] = 0$$

The above equation may be written as follows by using Eq. (8):

16 $$r^{2} \frac{{d^{2} P_{j} }}{{dr^{2} }} + r\,\frac{{dP_{j} }}{d\,r} - (k^{2} r^{2} + n^{2} )P_{j} = 0,$$

which is the modified Bessel equation, and its solution becomes

17 $$P_{1} (r) = C_{1} I_{n} (k\,r)\quad {\text{and}}\quad P_{2} (r) = B_{1} K_{n} (k\,r)$$

and hence, the complete solution of the pressure is:

18 $$p_{1} = C_{1} I_{n} (k\,r)\,Exp\,\left[ {i(kz + n\theta ) + \omega t} \right]$$

19 $${\text{and}}\quad p_{2} = B_{1} K_{n} (k\,r)\,Exp\,\left[ {i(kz + n\theta ) + \omega t} \right]$$

The integral constants $C_{1} \,$ and $\,B_{1}$ may be determined from the BCs, as demonstrated in the next section. Furthermore, $I_{n} (k\,r)\,\,{\text{and}}\,{\text{ K}}_{{\text{n}}} (k\,r)$ there are modified Bessel functions of the 1st and 2nd kinds. As shown in Eqs. (7) and (8) are substituted into Eqs. (11)–(13), the following differential equations are obtained:

20 $$r^{2} \frac{{d^{2} V_{rj} }}{{d\,r^{2} }} + r\,\frac{{d\,V_{rj} }}{dr} - (s_{1}^{2} r^{2} + n^{2} + 1)\,V_{rj} = \frac{{r^{2} \lambda \,}}{{\mu^{\prime}_{j} }}\frac{{dP_{j} }}{dr} + 2\,i\,n\,V_{\theta j} ,$$

21 $$r^{2} \frac{{d^{2} V_{\theta j} }}{{d\,r^{2} }} + r\,\frac{{d\,V_{\theta j} }}{dr} - (s_{1}^{2} r^{2} + n^{2} + 1)\,V_{\theta j} = \frac{\,inr\lambda \,}{{\mu^{\prime}_{j} }}P_{j} - 2\,i\,n\,V_{rj} ,$$

22 $$r^{2} \frac{{d^{2} V_{zj} }}{{d\,r^{2} }} + r\,\frac{{d\,V_{zj} }}{dr} - (s_{1}^{2} r^{2} + n^{2} )\,V_{zj} = \frac{{ikr^{2} \lambda \,}}{{\mu^{\prime}_{j} }}P_{j} ,$$

23 $${\text{where}}\quad s_{j}^{2} \,\, = k^{2} + \frac{{\mu_{j} + \lambda \,\rho_{j} (\omega + i\,k\,U_{j} )}}{{\mu^{\prime}_{j} }}$$

By solving the previous Eqs. (20)–(22) and using Eq. (10), one obtains the solutions as follows:

For the inner fluid (Liquid Phase)

24 $$v_{r1} = \left[ {\frac{{k\lambda \,C_{1} I^{\prime}_{n} (k\,r)}}{{\mu^{\prime}_{1} (k^{2} - s_{1}^{2} )}} - \frac{{i\,k\,C_{2} }}{{s_{1} }}I_{n - 1} (s_{1} r) + \frac{{n\,C_{3} }}{{s_{1} r}}I_{n} (s_{1} r)} \right]Exp\,\left[ {i(kz + n\theta ) + \omega t} \right],$$

25 $$v_{\theta 1} = i\left[ {\frac{{n\lambda \,C_{1} I_{n} (k\,r)}}{{\mu^{\prime}_{1} (k^{2} - s_{1}^{2} )}} - \frac{{i\,k\,C_{2} }}{{s_{1} }}I_{n - 1} (s_{1} r) + C_{3} I^{\prime}_{n} (s_{1} r)} \right]Exp\,\left[ {i(kz + n\theta ) + \omega t} \right],$$

26 $${\text{and}}\quad v_{z1} = \left[ {\frac{{i\,k\,\lambda \,C_{1} I_{n} (k\,r)}}{{\mu^{\prime}_{1} k^{2} - s_{1}^{2} )}} + C_{2} I_{n} (s_{1} r)} \right]Exp\,\left[ {i(kz + n\theta ) + \omega t} \right].$$

For the outer fluid (Gas Phase)

27 $$v_{r2} = \left[ {\frac{{k\lambda \,B_{1} K^{\prime}_{n} (k\,r)}}{{\mu^{\prime}_{2} (k^{2} - s_{2}^{2} )}} + \frac{{i\,k\,B_{2} }}{{s_{2} }}K_{n - 1} (s_{2} r) + \frac{{n\,B_{3} }}{{s_{2} r}}K_{n} (s_{2} r)} \right]Exp\,\left[ {i(kz + n\theta ) + \omega t} \right],$$

28 $$v_{\theta 2} = i\left[ {\frac{{n\lambda \,B_{1} K_{n} (k\,r)}}{{\mu^{\prime}_{2} (k^{2} - s_{2}^{2} )}} + \frac{{i\,k\,B_{2} }}{{s_{2} }}K_{n - 1} (s_{2} r) + B_{3} K^{\prime}_{n} (s_{2} r)} \right]Exp\,\left[ {i(kz + n\theta ) + \omega t} \right],$$

29 $${\text{and}}\quad v_{z2} = \left[ {\frac{{i\,k\,\lambda \,B_{1} K_{n} (k\,r)}}{{\mu^{\prime}_{2} (k^{2} - s_{2}^{2} )}} + B_{2} K_{n} (s_{2} r)} \right]Exp\,\left[ {i(kz + n\theta ) + \omega t} \right].$$

Correspondingly, by solving Eq. (6) and using Eq. (9), the solution of the EF takes the following form:

30 $$\psi_{1} = C_{4} I_{n} (k\,r)Exp\left[ {i(kz + n\theta ) + \omega t} \right],$$

31 $${\text{and}}\quad \psi_{2} = B_{4} K_{n} (k\,r)Exp\left[ {i(kz + n\theta ) + \omega t} \right].$$

where $C_{i} \,$ and $\,B_{i}$, $i = 1,\,2,\,3,\,4$ are the integrating constants that can be derived from the applicable BCs in the following section, and the prime represents the derivative concerning the argument.

## BCs and dispersion relation

The velocities and electric potential distributions are involved in the BCs and the stability hypothesis. These conditions can be classified at the perturbed interface at

32 $$\eta = \eta_{0} Exp\left[ {i(kz + n\theta ) + \omega t} \right].$$

where $\eta_{0}$ is the surface's initial amplitude.

- The kinematic BC gives^44^:
  33 $$\frac{dS}{{dt}} = 0\,\, \Rightarrow v_{rj} = \frac{\partial \eta }{{\partial t}} + U_{j} \frac{\partial \eta }{{\partial z}}\quad {\text{at}}\quad r = R + \eta .$$
  where $S = r - R - \eta$ gives the equation of the disturbed surface.
- The continuity of speed at the separation surface gives^44^:
  34 $$v_{\theta 1} = v_{\theta 2} \quad {\text{at}}\quad r = R + \eta$$
  35 $${\text{and}}\quad v_{z1} = v_{z2} \quad {\text{at}}\quad r = R + \eta$$
  The stress tensor in case of the couple-stress model takes the form^16,45^
  36 $$\tau_{ik} = - p\delta_{ik} + \left( {\mu - \mu^{\prime}\,\nabla^{2} } \right)\,\left( {\frac{{\partial v_{i} }}{{\partial x_{k} }} + \frac{{\partial v_{k} }}{{\partial x_{i} }}} \right) + \varepsilon E_{i} E_{k} - \frac{1}{2}\varepsilon \delta_{ik} E^{2}$$
  where $\delta_{ik}$ is the Kronecker delta. Hence, the stress tensor's tangential part should be continuous at the interface, leading to the following BCs:
- The shear stresses must be continuous at the surface of the separation
  37 $$\tau_{rz}^{(1)} = \tau_{rz}^{(2)} \Rightarrow \left( {\mu_{1} - \mu^{\prime}_{1} \nabla^{2} } \right)\left( {\frac{{\partial v_{r1} }}{\partial z} + \frac{{\partial_{z1} }}{\partial r}} \right) = \left( {\mu_{2} - \mu^{\prime}_{2} \nabla^{2} } \right)\left( {\frac{{\partial v_{r2} }}{\partial z} + \frac{{\partial v_{z2} }}{\partial r}} \right)\quad {\text{at}}\quad r = R + \eta$$
  and
  38 $$\tau_{r\theta }^{(1)} = \tau_{r\theta }^{(2)} \Rightarrow \left( {\mu_{1} - \mu^{\prime}_{1} \nabla^{2} } \right)\left( {\frac{{\partial v_{\theta 1} }}{\partial r} - \frac{{v_{\theta 1} }}{r} + \frac{1}{r}\frac{{\partial v_{r1} }}{\partial \theta }} \right) = \left( {\mu_{2} - \mu^{\prime}_{2} \nabla^{2} } \right)\left( {\frac{{\partial v_{\theta 2} }}{\partial r} - \frac{{v_{\theta 2} }}{r} + \frac{1}{r}\frac{{\partial v_{r2} }}{\partial \theta }} \right)\quad {\text{at}}\quad r = R + \eta$$
  Following the BCs used before by Refs.^14^ for the electrostatic field, one discovers.
- The tangential part of EF is continuous at the separation surface
  39 $$\underline{N} \times \underline{E}_{1} = \underline{N} \times \underline{E}_{2} \Rightarrow \,\,\,\,\,\psi_{1} = \psi_{2} \quad {\text{at}}\quad r = R + \eta$$
- The normal part of EF is continuous at the surface of separation, i.e.,
  40 $$\underline{N} \,.\left( {\varepsilon_{1} \underline{E}_{1} } \right) = \underline{N} \,.\left( {\varepsilon_{2} \underline{E}_{2} } \right) \Rightarrow \varepsilon_{1} \,\frac{{\partial \psi_{1} }}{dr} - \,\varepsilon_{2} \,\frac{{\partial \psi_{2} }}{dr} + ikE_{0} \eta (\varepsilon_{1} - \varepsilon_{2} ) = 0\quad {\text{at}}\quad r = R + \eta$$
  where $\underline{N} = \frac{\nabla S}{{\left| {\nabla S} \right|}}$ is the unit vector normal to the perturbed surface.

Substituting from Eqs. (24)–(31) into the BCs in Eqs. (33)–(40), one gets

41 $$C_{1} = \frac{{\sqrt {\rho_{1} \sigma R} }}{R}\left[ {(\omega + i\,k\,U_{1} )\frac{{\Delta_{11} }}{\Delta } - (\omega + i\,k\,U_{2} )\frac{{\Delta_{12} }}{\Delta }} \right]\eta_{0}$$

42 $$C_{2} = \left[ { - (\omega + i\,k\,U_{1} )\frac{{\Delta_{21} }}{\Delta } + (\omega + i\,k\,U_{2} )\frac{{\Delta_{22} }}{\Delta }} \right]\eta_{0}$$

43 $$C_{3} = \left[ {(\omega + i\,k\,U_{1} )\frac{{\Delta_{31} }}{\Delta } - (\omega + i\,k\,U_{2} )\frac{{\Delta_{32} }}{\Delta }} \right]\eta_{0}$$

44 $$B_{1} = \frac{{\sqrt {\rho_{1} \sigma R} }}{R}\left[ { - (\omega + i\,k\,U_{1} )\frac{{\Delta_{41} }}{\Delta } + (\omega + i\,k\,U_{2} )\frac{{\Delta_{42} }}{\Delta }} \right]\eta_{0}$$

45 $$B_{2} = \left[ {(\omega + i\,k\,U_{1} )\frac{{\Delta_{51} }}{\Delta } - (\omega + i\,k\,U_{2} )\frac{{\Delta_{52} }}{\Delta }} \right]\eta_{0}$$

46 $$B_{3} = \left[ { - (\omega + i\,k\,U_{1} )\frac{{\Delta_{61} }}{\Delta } + (\omega + i\,k\,U_{2} )\frac{{\Delta_{62} }}{\Delta }} \right]\eta_{0}$$

47 $$C_{4} = \frac{{ - i\,E_{0} \eta_{0} (\varepsilon_{1} - \varepsilon_{2} )\,K_{n} (kR)}}{{\varepsilon_{1} I^{\prime}_{n} (kR)\,K_{n} (kR) - \varepsilon_{2} I_{n} (kR)\,K^{\prime}_{n} (kR)}}$$

48 $$B_{4} = \frac{{ - i\,E_{0} \eta_{0} (\varepsilon_{1} - \varepsilon_{2} )\,I_{n} (kR)}}{{\varepsilon_{1} I^{\prime}_{n} (kR)\,K_{n} (kR) - \varepsilon_{2} I^{\prime}_{n} (kR)\,K^{\prime}_{n} (kR)}}$$

where $\Delta ,\,\,\Delta_{11} \,,...,\Delta_{62} ,$ are listed in the ESM Appendix, and $\sigma$ refers to the ST.

Additionally, the normal stress BC is determined by^12–14,16,44^:

49 $$\left( {\tau_{ik}^{(1)} - \tau_{ik}^{(2)} } \right)\,.\,\underline{n} + \sigma \nabla .\,\underline{N} = 0\quad {\text{at}}\quad r = R + \eta ,$$

then we have the following condition to the first order terms^12–14,16,44^:

50 $$p_{1} - p_{2} - 2(\mu_{1} - \mu^{\prime}_{1} \,\nabla^{2} )\frac{{\partial v_{r1} }}{\partial r} + 2(\mu_{2} - \mu^{\prime}_{2} \,\nabla^{2} )\frac{{\partial v_{r2} }}{\partial r} - E_{0} \left( {\varepsilon_{1} \frac{{\partial \psi_{1} }}{\partial z} - \varepsilon_{2} \frac{{\partial \psi_{2} }}{\partial z}} \right) + \frac{\sigma }{{R^{2} }}\left( {1 + R^{2} \frac{{\partial^{2} }}{{\partial z^{2} }} + \frac{{\partial^{2} }}{{\partial \theta^{2} }}} \right)\eta = 0.$$

Substituting Eqs. (18), (19), (24), (27), and (30–32) into Eq. (50) yields the following dispersion relationship between $k^{*}$ and $\omega^{*}$

51 $$\left( {\omega + i\,k\,U_{1} } \right)\delta_{1} - \left( {\omega + i\,k\,U_{2} } \right)\delta_{2} - \frac{{k\,R\,E^{2} (\varepsilon_{1} - \varepsilon_{2} )^{2} I_{n} (kR)\,K_{n} (kR)}}{{\sqrt {\rho_{1} \sigma \,R} \left[ {\varepsilon {}_{1}I^{\prime}_{n} (kR)\,K_{n} (kR) - \varepsilon_{2} I_{n} (kR)K^{\prime}_{n} (kR)} \right]}} + \frac{\sigma }{{R\sqrt {\rho_{1} \sigma \,R} }}(1 - k^{2} R^{2} - n^{2} ) = 0,$$

where $\delta_{1} ,\,\delta_{2}$ are listed in the ESM Appendix.

Equation (51) can be written in a dimensionless formula as

52 $$\left( {\omega^{*} + \,i\,k^{*} \,\sqrt {we} } \right)\delta_{1} - \left( {\omega^{*} + i\,k^{*} \,\tilde{U}\,\sqrt {we} } \right)\delta_{2} - \frac{{k^{*} \,\,E_{0}^{*2} (1 - \tilde{\varepsilon })^{2} I_{n} (k^{*} )\,K_{n} (k^{*} )}}{{\left[ {I^{\prime}_{n} (k^{*} )\,K_{n} (k^{*} ) - \tilde{\varepsilon }\,I_{n} (k^{*} )K^{\prime}_{n} (k^{*} )} \right]}} + (1 - k^{*2} - n^{2} ) = 0$$

where

53 $$s_{1}^{*2} = k^{*2} + \frac{{Da(\omega^{*} + i\,k^{*} \,\sqrt {we} ) + z}}{z\Lambda },$$

54 $$s_{2}^{*2} = k^{*2} + \frac{{\tilde{\rho }\,Da(\omega^{*} + i\,k^{*} \,\tilde{U}\sqrt {we} ) + \tilde{\mu }\,z}}{{\tilde{\mu }^{\prime}z\Lambda }}$$

where $\omega^{*} = \omega_{r}^{*} + i\sqrt {We} \omega_{i}^{*}$, $\omega^{*} = \omega \sqrt {\rho_{1} R^{3} /\sigma }$ is a dimensionless growth level, $\omega_{i}^{*} = \left( {R/U_{1} } \right)\omega_{i}$ is a disrupted frequency with no dimensions, $k^{*} = kR$ is the non-dimensional wave numeral, $s_{j}^{*} = s_{j} R$, $\tilde{\rho } = \rho_{2} /\rho_{1}$ is the density ratio, $\tilde{U} = U_{2} /U_{1}$ is the velocity ratio, $\tilde{\mu } = \mu_{2} /\mu_{1}$ is the viscosity ratio, $\tilde{\mu }^{\prime} = \mu^{\prime}_{2} /\mu^{\prime}_{1}$ is the viscoelasticity ratio, $\tilde{\varepsilon } = \varepsilon_{2} /\varepsilon_{1}$ is the ratio of the dielectric constant, $Da = \lambda /R^{2}$ is the Darcy number, $E_{0}^{*} = E_{0} \sqrt {\varepsilon_{1} R/\sigma }$ is the non-dimensional electric field, $We = \rho_{1} U_{1}^{2} R/\sigma$ is the liquid Weber number, $z = \mu_{1} /\sqrt {\rho_{1} \sigma \,R}$ is the Ohnesorge number, $\Lambda = \mu^{\prime}_{1} /\mu R^{2}$ is the couple-stress parameter.

The calculations that follow mostly use Gaster’s technique^46^ to assess the impact of physical factors on the instability profile. Therefore, one may confirm these elements by applying Gaster's approach^46^ and Mathematica Software (MS) Version 12.0.0.0 as a mathematical instrument.

The stability analysis at this point is dependent on the dispersion equation provided in Eq. (52). It truly lacks a precise solution. For this equation, numerical computations will be made. The next section provides examples of this process; for further information, see^38,43,45^.

## Discussions of outcomes

**Figure 2:**
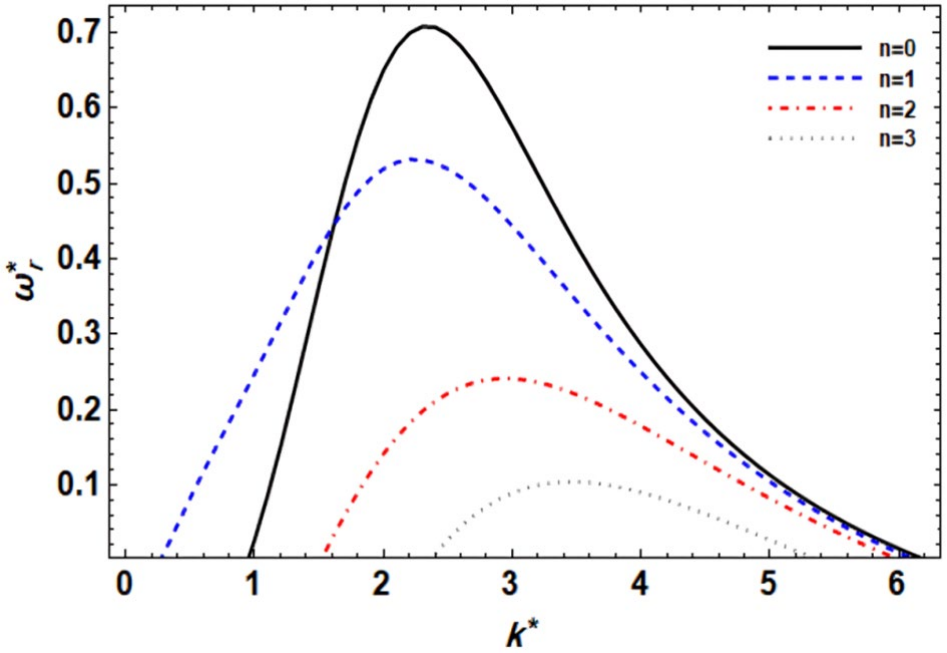
displays the growth level vs. the wave numeral of different values of $n$.

**Figure 3:**
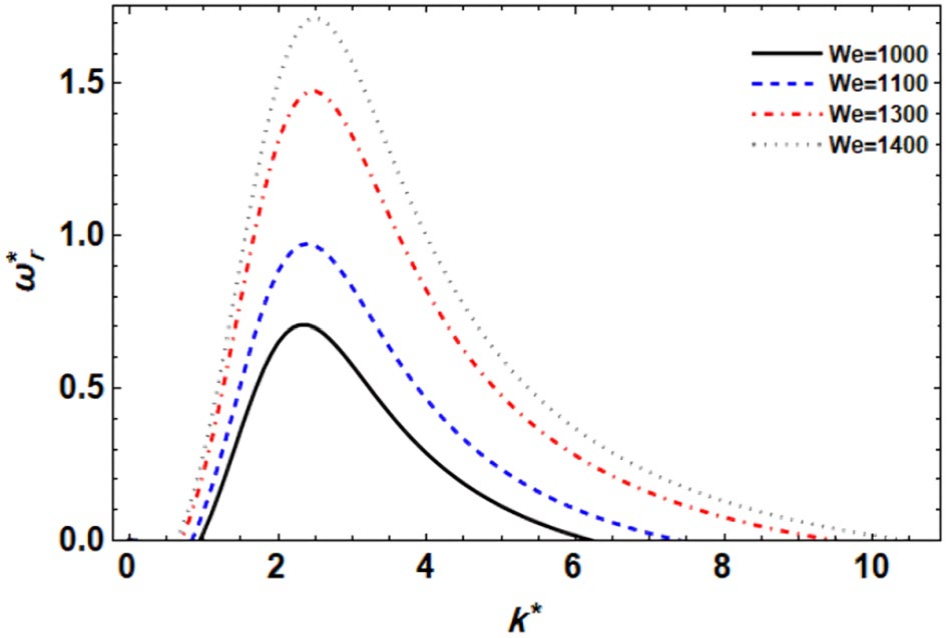
displays the growth level vs. the wave numeral of different values of $We$.

**Figure 4:**
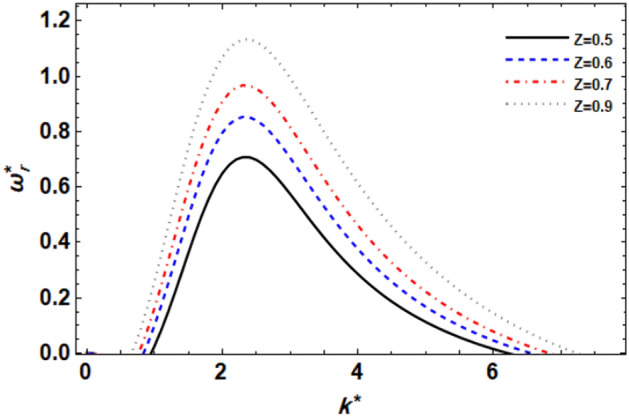
displays the growth level vs. the wave numeral of different values of $Z$.

**Figure 5:**
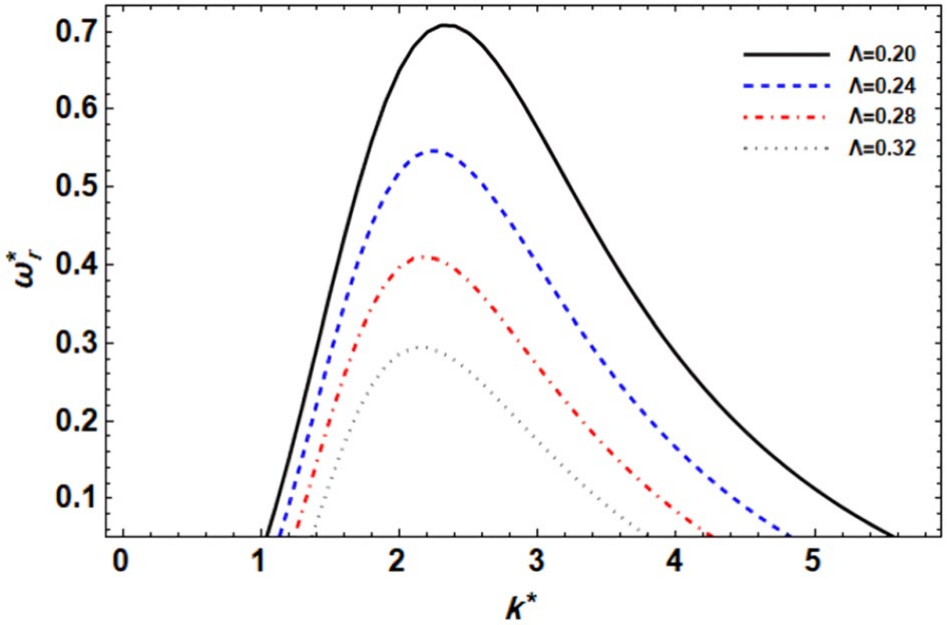
displays the growth level vs. the wave numeral of different values of $\Lambda$.

**Figure 6:**
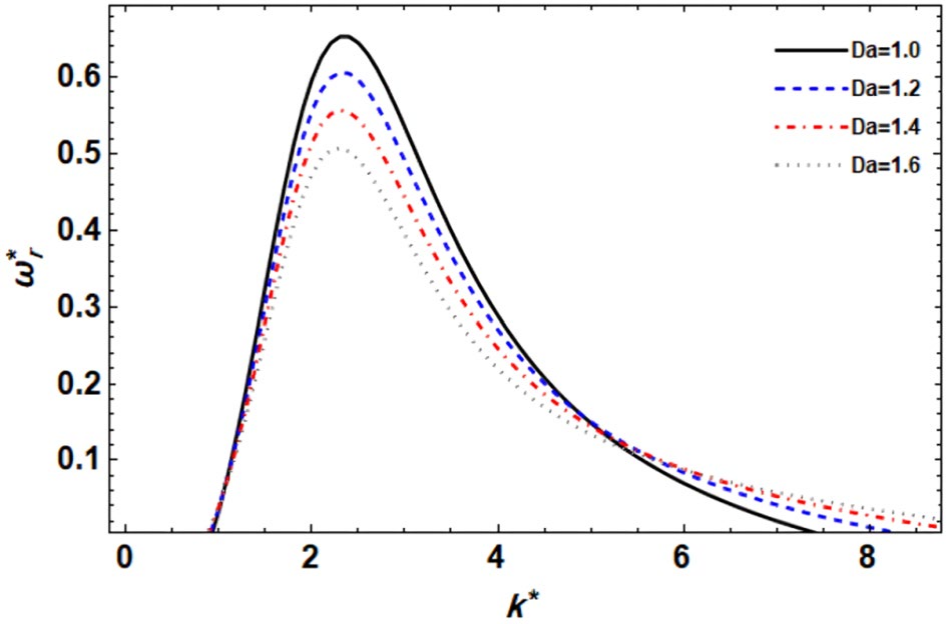
displays the growth level vs. the wave numeral of different values of $Da$.

**Figure 7:**
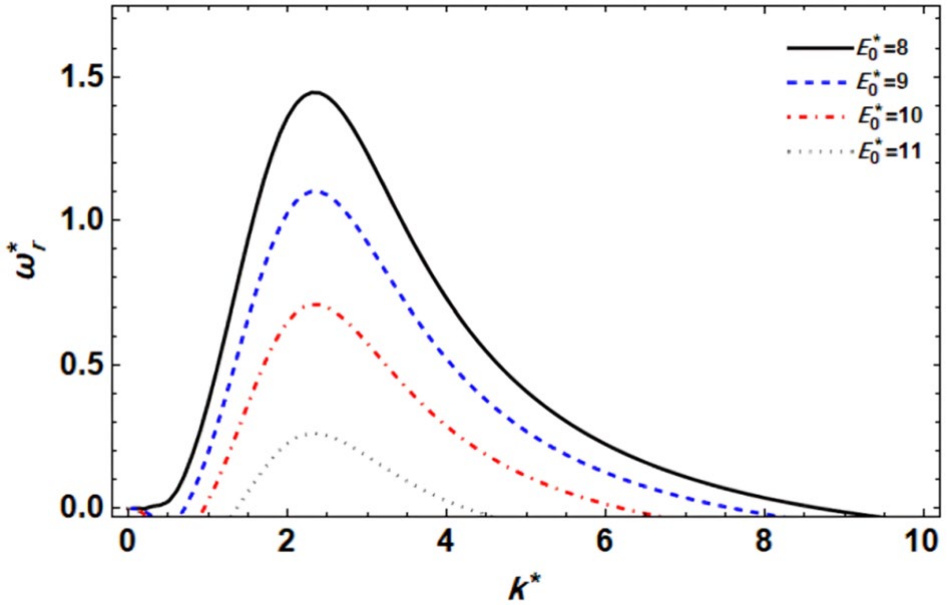
displays the growth level vs. the wave numeral of different values of $E_{0}^{*}$.

**Figure 8:**
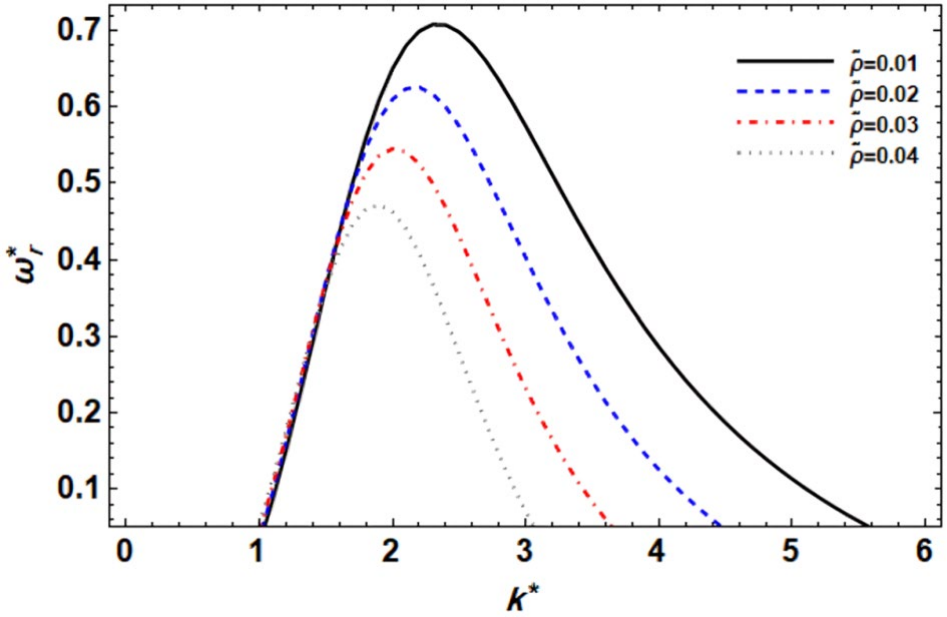
displays the growth level vs. the wave numeral of different values of $\tilde{\rho }$.

**Figure 9:**
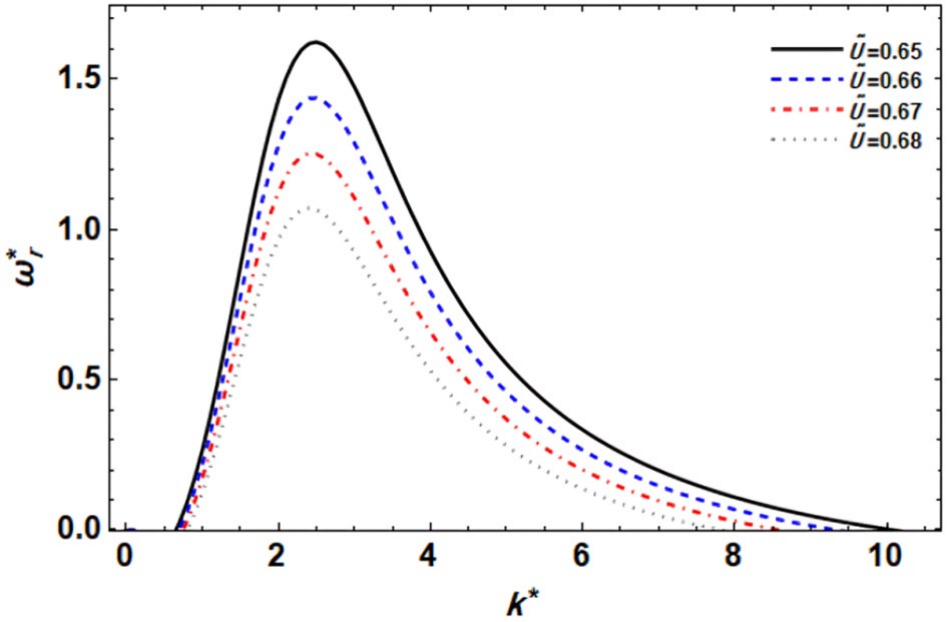
displays the growth level vs. the wave numeral of different values of $\tilde{U}$.

**Figure 10:**
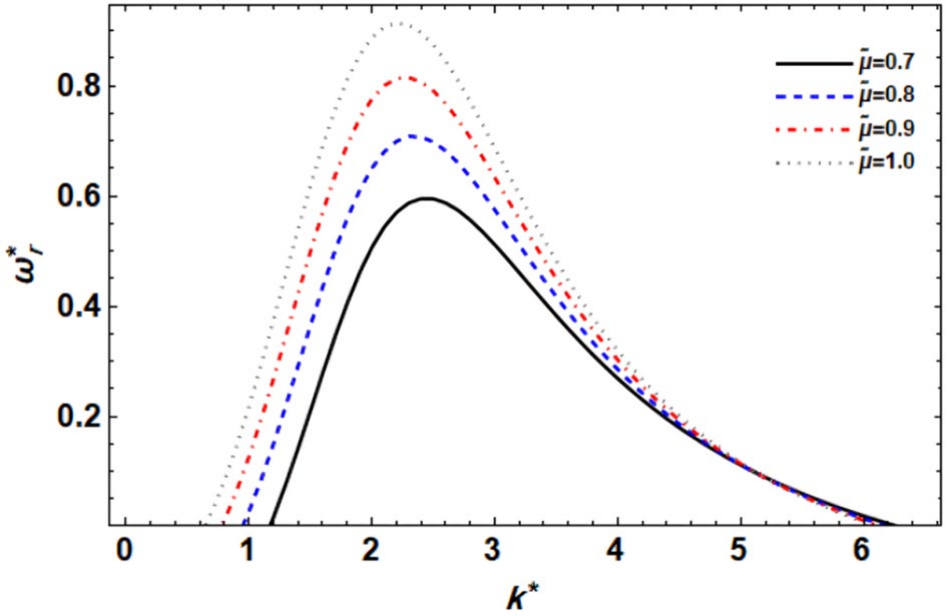
displays the growth level vs. the wave numeral of different values of $\tilde{\mu }$.

**Figure 11:**
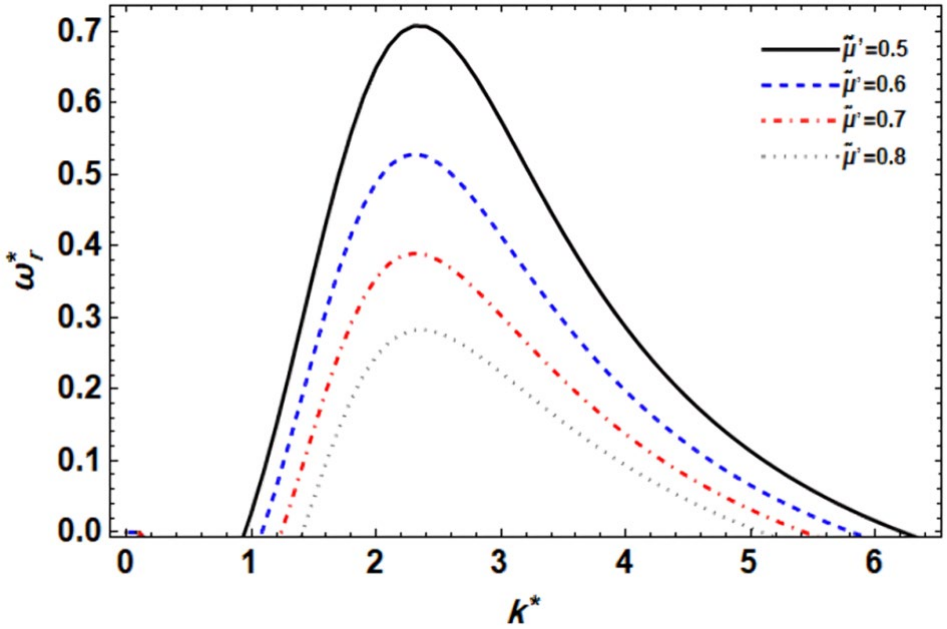
displays the growth level vs. the wave numeral of different values of $\tilde{\mu^{\prime}}$.

As previously demonstrated, the dimensionless dispersion relationship is formed as given in Eq. (52). The angular frequency of waves is often of complex form in the backdrop of the time-dependent instability analysis, where the real part denotes the disruption growth level and the imaginary part signifies the disruption frequency. The system becomes unstable when the growth level is positive. It is not certain that a closed formula of the analytic solution will be achieved. Consequently, the MS can be used to adjust a numerical method. Equivalent outcomes have been made available^45,47^. In a vision of the Gaster way^46^, one may put $\omega_{i}^{*} = - k^{*}$, and take $\omega_{r}^{*} = 1000$ by mode of a primary estimate the solution. A recurrence of the ordered pair solution $(k^{*} ,\omega_{r}^{*} )$ at various criteria of dissimilar factors is included in this examination. The next style displays a sequence of graphs for the relevant study that range from Figs. 2, 3, 4, 5, 6, 7, 8, 9, 10 and 11. Over these graphs, growth levels are drawn vs. the wave numeral of the waves. For more simplicity, the next factors are selected:

$$\,We = 1000,\,Z = 0.5,\,\Lambda = 0.2,\,\,Da = 0.7,\,\,\tilde{\rho } = 0.01,\,\tilde{\mu } = 0.8,\,\tilde{\mu^{\prime}} = 0.5,\,\tilde{U} = 0.7,\,\tilde{\varepsilon } = 0.5,\,E_{0}^{*} = 10,\,\,\,{\text{and}}\,\,\,n = 0$$

### ***The effect of the azimuthal wave numeral ***$n$

Figure 2 shows the relation between $\omega_{r}^{*}$ and $k^{*}$ for dissimilar modes of disturbances $n$. By adjusting the azimuthal wave number ($n$), it is possible to control the flow dynamics and stability characteristics of the liquid jet. For example, selecting an appropriate $n$ value may help mitigate instabilities and promote a more stable and controlled flow behavior. It is clear that for $n = 1$ (asymmetric mode) the jet is more unstable than the other modes for a small wave number (actually at $k^{*}$ ≤ 1.5 for this particular case). Except for this variance, all the outcomes displayed have proven the general ideal theorem that 2-dimensional ($n = 0$) disruptions are more unstable than 3-dimensional ones, as predicted by Squire’s theorem^48^. Nonetheless, for viscid and viscoelastic liquid jets, Squire’s theory is no longer effective. In addition, it is noticed from this figure that the extreme growth level becomes smaller as $n$ increases, while the dominant wave numbers increase except as $n = 1$. And the growth level of 2-dimensional surpasses those of 3-dimensional in the wave numerals range 1.5 ≤ $k^{*}$ ≤ 6. These results agree with those obtained earlier^43^. In addition, experimental studies can be conducted to validate the influence of $n$ on flow stability in liquid jets. Observations of flow patterns and disturbance growth rates can provide insights into the relationship between azimuthal wave number and flow stability, helping to refine theoretical models and predictive capabilities.

### ***Impact of Weber numeral ***$We$

Figure 3 displays the relationship between $\omega_{r}^{*}$ and $k^{*}$ of series values of the liquid Weber numeral for ($n = 0$). In the context of flow stability, the Weber number plays a significant role in determining the behavior of the flow and its stability characteristics. It is evident that as the We rises, the growth levels seem weak until the wave numeral of $k^{*} = 2$. Then, the instability power implies that the extra growth level and the central wave numerals also rise. The development of the Weber numeral may be made by improving the liquid density and velocity, or by reducing the ST. Furthermore, as the liquid Weber numeral rises, the destabilizing zone also rises. So, the Weber numeral has instability power. These outcomes are well-matched with the results of the previous studies^49^.

### ***Impact of Ohnesorge numeral ***$Z$

The viscosity effects of the porous media on the amount of disturbance growth are shown in Fig. 4. It is evident that when the Ohnesorge numeral grows, so does the growth level, which increases the instability zone. This demonstrates that the stability image is destabilized by the Ohnesorge numeral Z. It is important to remember that the Ohnesorge number represents the ratio of the viscous strength to the ST strength, and thus the lower Ohnesorge numeral results in a lower viscous force in contrast to the ST force. The growth level is smaller in this situation. One may claim that when the Ohnesorge numeral rises, the maximal growth also does. The Ohnesorge number normally has stabilizing effects because of viscosity, however, El-Sayed et al.^44^ have proven that the Ohnesorge numeral also shows a dual function by stabilizing and then destabilizing the stability picture. Also, keep in mind that the viscosity in this case is due to the porous media rather than the fluid's characteristics, therefore its impact is weakened compared to that of the fluid's viscosity. In summary, the Ohnesorge number plays a crucial role in determining flow stability, influencing the balance between viscous and surface tension forces. Higher Ohnesorge numbers are associated with increased instability, contributing to a wider range of flow conditions that promote flow destabilization. The identical outcomes have already been verified^47^.

### ***Impact of the CSF parameter ***$\Lambda$

Figure 5 explains the viscoelastic effects resulting from the CSFs on the disruption growth level. The viscoelastic effects resulting from the couple stress fluid parameter impact the flow dynamics by introducing additional internal forces within the fluid. These internal forces counteract the growth of disturbances, leading to a stabilization of the flow. It is also evident that when the couple-stress parameter increases, the growth level drops as well, reducing the instability zone. This shows that the couple-stress parameter stabilizes the system under consideration. Kumara Shiva et al.^50^ established the same result. Mathematically, it is worth noting that if we look at the equation of motion in Eq. (2), one can observe that the viscoelasticity $\mu^{\prime}$ is the dynamic viscosity in the original Navier–Stokes equation. Therefore, in the original research of the viscid liquid jet, the influence of this non-dimensional parameter (couple-stress parameters $\Lambda$) is equivalent to the effect of viscosity; for example, see^37,45^. This result also concurs with Kumar^51^.

### ***Effect of the Darcy numeral ***$Da$

The effect of permeability on the stability picture throughout the Darcy numeral Da is seen in Fig. 6. It is clear that increasing Da minimizes the level of growth interruptions, and as a result, the unstable area shrinks dramatically. Conversely, in the unstable region, huge wave numerals result in a little destabilizing influence. The stability process is made more unstable by the Darcy number Da which agrees with Refs.^14,45^. According to Ref.^52^, Da has a stabilizing impact. As a result, the parameter Da gains twofold relevance. Furthermore, when Da grows, the central and lower cut-off wave numerals remain constant, but the higher cut-off wave numbers rise. Nonetheless, when Da grows, so does the maximum growth level. On the other hand, in porous media flows, such as those encountered in fiber-reinforced composite materials, the Darcy number plays a crucial role in determining the flow characteristics. It governs the flow resistance within the porous structure and affects the distribution of fluid velocities and pressures.

### ***Effect of the EF ***$E_{0}^{*}$

In the case of a 2-dimensional setup, Fig. 7 displays the relationship between $\omega_{r}^{*}$ and $k^{*}$ for numerous values of the EF factor. From this graph, it can be shown that the effect of the EF factor is the same as the CSF parameter. Additionally, it is evident that the values of the instability zone and the growth level of disruptions significantly decrease when $E_{0}^{*}$ is raised. Additionally, as the EF parameter increases, the dominating wave numerals, upper cut-off, and maximal growth level all drop, while the wave numerals for the lower cut-off rise. Therefore, it may be said that the current system is stabilized by the applied EF. Many studies have already confirmed this result. For example, it agrees with the results reported in Ref.^53^. On the other hand, the tangential electric field applies EHD forces to the fluid, which can alter the flow behavior. These forces arise from how the electric field and the charges present in the fluid, leading to the generation of electrical stresses and potentially affecting the fluid flow. Electrokinetic events can also arise due to the existence of a tangential electric field, such as electroosmosis or electrophoresis, which further influences the flow stability. These phenomena involve the movement of charged particles or fluids when the electric field is at work and can significantly impact the flow behavior.

### ***Effect of the Gas-to-Liquid (***GTL***) density relationship ***$\tilde{\rho }$

The role of the GTL density relationship $\tilde{\rho }$ is demonstrated in Fig. 8. The curves have been produced for serial values of $\tilde{\rho }$ as can be seen in this graph. According to this graph, increasing the GTL density ratio has a very mild destabilizing impact on tiny wave numbers (actually, $k^{*}$ ≤ 1.4 for this particular case). The impact is then mirrored to improve the stability zone. Furthermore, raising the GTL density ratio values results in a decrease in the maximal growth level, central, and upper cut-off wave numeral. By contrast, the lower cut-off wave numeral remains constant by increasing the GTL density ratio. The instability zone significantly shrinks as a result of the prior reasoning. This demonstrates that concerning the VPT in the permeable medium, the GTL ratio of density $\tilde{\rho }$ has a stabilizing impact. It is worth noting that this factor has a dual purpose in the context of the linear stability study. This outcome is consistent with the previously verified result by^14^. Correspondingly, changes in the GTL density relationship can significantly impact flow dynamics and stability. Alterations in the density relationship may arise from variations in gas and liquid properties, temperature, or pressure conditions, all of which can affect the stability characteristics of the flow system. As illustrated in Figs. 5 and 8, the couple-stress parameter and the GTL density relationship have a stabilizing impact for higher values of the wave numerals. This effect is caused by a rise in the viscoelasticity and inertia of CSF dampening. As a result, a rise in viscoelasticity and the GTL density ratio makes the free surface more stable.

### ***Effect of the GTL velocity ratio ***$\tilde{U}$

Understanding the GTL velocity relationship is crucial in various engineering applications involving liquid jets, such as fuel injection systems, chemical spray nozzles, and inkjet printing. Controlling the relative velocities between the gas and liquid phases is essential for optimizing the stability and performance of these systems. The impression of the GTL velocity relationship $\tilde{U}$ for axisymmetric disruptions as seen in Fig. 9 must be explained. It makes sense that when the GTL velocity ratio $\tilde{U}$ increases, the disruption growth level and the unstable zone fall sharply, and the maximal growth level and the unstable region also drop. This indicates that the interface is stabilized by the GTL velocity ratio. Additionally, the central and higher cut-off wave numerals are both decreased by the growth of the GTL velocity ratio, whereas the lower cut-off wave numeral is somewhat increased. Overall, it can be said that the GTL velocity relationship opposes the atomization procedure. The ambient gas velocity is responsible for this influence because the Weber numeral is of a fixed value in this location, meaning that it has a stabilizing effect. Similar outcomes have recently been discovered^49^.

### ***Effect of the GTL dynamic viscosity ratio ***$\tilde{\mu }$

Figure 10 illustrates how the GTL dynamic viscosity ratio affects the growth level disruptions for a series of these parameter values. This graph clearly shows that when the GTL dynamic viscosity ratio rises, both the maximal growth level and the instability zone expand until a critical wave numeral, in this case, reaches $k^{*} = 5$. After that, the influence is redirected to a weakly stabilizing effect. By contrast, both the center wave numeral and the lower cut-off wave numeral decrease. This displays that the GTL dynamic viscosity ratio has a double effect on stability, first destabilizing and then stabilizing. It is worth noting that because the liquid Ohnesorge numeral is set here, this effect is attributable to the viscosity of the surrounding gas, i.e., the surrounding gas viscosity caused by the porous material has a dual effect on the system at issue. In applications such as fuel injection systems or spray coating processes, the viscosity ratio plays a crucial role in determining the atomization behavior of the liquid jet. A higher viscosity ratio can lead to finer atomization and improved spray characteristics due to the enhanced stability of the liquid jet. Comparable results have recently been discovered^47^.

### ***Impact of the GTL viscoelasticity ratio ***$\tilde{\mu^{\prime}}$

To explore the impacts of the GTL viscoelasticity ratio $\tilde{\mu^{\prime}}$ on the stability requirements, four various values of $\tilde{\mu^{\prime}}$ are gathered in Fig. 11. Because the instability occurs from the positive sign of the real component of the frequency, we can see in this figure that increasing the GTL viscoelasticity ratio reduces the instability zone and so stabilizes the liquid jet. It has also been discovered that when the GTL viscoelasticity ratio grows, the maximal growth level, the dominating, and greater cut-off wave numerals rise, whereas raising the GTL viscoelasticity ratio raises the lower cut-off wave numbers. Because the couple-stress parameters are set here, this influence is attributable to the surrounding gas viscoelasticity, i.e., the CSF-induced surrounding gas viscosity has a stabilizing effect on the considered system. Understanding the influence of the GTL viscoelasticity ratio on flow stability is crucial in various engineering applications, such as atomization processes, coating technologies, and fuel injection systems. Optimizing the GTL viscoelasticity ratio can help control and enhance the stability of liquid jets, leading to improved performance and efficiency in these applications.

## Concluding remarks

The CSF framework describes axisymmetric and asymmetric streaming flows, which are examined in this article. The CSF is a liquid that has been implanted with microfibers, similar to fiber-reinforced composite substances. It is a mechanism that divides the two CSF structures with an unending vertical cylindrical interface. The study's impetus is explained as stemming from the CSFs' increasing importance in contemporary industry and technology, specifically in the creation of fiber-reinforced composite materials. In addition to the effect of CSF, an axial EF is applied across the cylindrical contact. To reduce mathematical complexity, the VPT is used for convenience. The main step in the linear technique is to combine the basic linear equations of motion with the appropriate linear-related BCs. A non-dimensional procedure generates a set of physically dimensionless numbers. The conditions for hypothetical linear stability are then worked out. The MS is used to calculate the dispersion relationships with the help of Gaster's theorem. It has been demonstrated that the presence of a porous material makes the system more unstable than it would be in the absence of one, after carefully studying several influences on the stability investigation of the system in question. More instability results in the axisymmetric disturbance situation. Several graphs show the linear approaches. It is discovered that increasing the gas-to-liquid viscoelasticity ratio, axial EF, and couple-stress parameters causes the system to become more stable. As the Weber and Ohnesorge numbers increase, the system may become unstable. Based on the critical wave number, the gas-to-liquid dynamic viscosity connection affects the system's stability or instability. This means that the Darcy number can stabilize or destabilize the system based on specific conditions. Understanding these properties' effects on stability is crucial for a variety of engineering applications and technological breakthroughs.

A variety of graphs have demonstrated the linear approaches. Therefore, the main keys to the outcomes may be listed as follows.

- The parameters of the azimuthal wave numeral $n$, couple-stress parameters $\Lambda$, EF $E_{0}^{*}$, GTL density ratio $\tilde{\rho }$, GTL velocity ratio $\tilde{U}$, and GTL viscoelasticity ratio $\tilde{\mu^{\prime}}$ have a stabilizing impact on the stability profile.
- The Weber numeral and Ohnesorge numeral Z parameters destabilize the system.
- The GTL dynamic viscosity relation has a twofold purpose in the instability image, separating instability and stability from the crucial wave number. Furthermore, the Darcy numeral Da serves a converse dual role in that it both stabilizes and destabilizes the system at issue.

## Here are some additional specific applications of the CSFs

The CSFs are a particular kind of non-Newtonian fluid that show more microstructural effects than what conventional Newtonian fluid models can capture. These extra effects include the existence of internal couples or moments in the fluid, which results from material rotations or microstructural asymmetries. Potential uses of the CSFs in a range of physical structures where these microstructural effects are important have been investigated. In what follows, some sources demonstrate the applicability and significance of CSF models in many scientific and technical domains by offering instances of its application to particular physical systems and phenomena:

- **Microscale flows in biological systems**
  It was discovered in simulating blood flow in microcirculation, where non-Newtonian behavior might result from relationships with the walls of vessels and red blood cell deformation^54^.
- **Emulsions and suspensions**
  It was found in investigating the stability and rheological characteristics of saturated formulations and suspensions, where flow behavior can be greatly influenced by internal microstructural influences^55^.
- **Polymer processing**
  It was discovered by comprehending the viscoelastic polymer mixtures' flow behavior and processing properties in extrusion and injection procedures^56^.
- **Microfluidic device**
  It was used to create and refine microfluidic instruments for use in biomedical settings where exact combining and fluid flow manipulation are crucial^57^.
- **Soft matter rheology**
  It was found in examining the rheological characteristics of complex soft matter systems under shear and extensional deformations, such as gels and liquid crystals^58^.

### Supplementary Information

Supplementary Information.

## Data Availability

All data generated or analyzed during this study are included in this manuscript and its supplementary information files.

## English symbols

$$(r,\theta ,z)$$: Cylindrical coordinates (m)
$U_{1}$: Initial liquid velocity (m s^−^^1^)
$U_{2}$: Initial gas velocity (m s^−^^1^)
$R$: The unperturbed radius of the jet (m)
$E_{0}$: Initial electric field (EF) (Newton/Coulomb)
$F(r)$: Amplitude of the initial disturbance
$c.\,c.$: Complex conjugate of the preceding term
$$k$$: Wave numeral (m^−^^1^)
$n$: Azimuthal wave number
$$v_{j}$$: Perturbed velocity (m s^−^^1^)
$\psi_{j}$: Perturbed electric potential (Newton m^−^^2^)
$p_{j}$: Perturbed pressure (Newton m^−^^2^)
$$g$$: Gravitational acceleration (m s^−^^1^)
$\underline{E}$: The intensity of the EF (Newton/Coulomb)
$$C_{1} ,\,C_{2} ,\,C_{3} ,C_{4}$$$$B_{1} ,\,B_{2} ,\,B_{3} ,B_{4}$$: Constants of integration
$\psi_{0j}$: Initial electric potential (Newton m^−^^2^)
$p_{0j}$: Initial pressure (Newton m^−2^)

## Greek symbols

$\rho_{j}$: Densities of two fluids (kg m^−^^3^)
$\mu_{j}$: Dynamic viscosities of two fluids (kg m^−^^3^ s^−^^1^)
$\sigma$: Surface tension (ST) (Newton m^−^^1^)
$\mu_{j}{\prime}$: Viscoelasticity of CSFs (kg m^−^^3^ s^−^^1^)
$$\lambda$$: Permeability (m^2^)
$\omega$: Frequency of the surface wave (s^−^^1^)
$\omega_{r}$: Amount of real growth rate (s^−^^1^)
$\omega_{i}$: Amount of imaginary growth rate (s^−^^1^)
$$\eta$$: Interfacial displacement (m)
$\eta_{0}$: The initial amplitude of the interfacial displacement (m)
$$\varepsilon_{j}$$: Dielectric constants

## Subscript

$$j=1$$: Liquid media
$$j=2$$: Gas media
