# Supplementary material for: EHD instability of a cylindrical interface separating two couple-stress fluids
Source: Sci Rep. 2024 Mar 7;14:5686. doi: 10.1038/s41598-024-56143-w (PMC11319821; doi:10.1038/s41598-024-56143-w)
Supplement: Supplementary file 1 — Supplementary Information. [file 41598_2024_56143_MOESM1_ESM.docx]

**Appendix**

The quantities utilized in Eq. (52) are listed below.

$\delta_{1}=\frac{\Delta_{11}\gamma_{1}}{\Delta}+\frac{ \Delta_{41}\gamma_{2}}{\Delta}-\frac{2i \Delta_{21}\gamma_{3}}{\Delta}+\frac{2i \Delta_{51}\gamma_{4}}{\Delta}-\frac{2 n\Delta_{31}\gamma_{5}}{\Delta}-\frac{2n \Delta_{61}\gamma_{6}}{\Delta}$,

$$\delta_{2}=\frac{\Delta_{12}\gamma_{1}}{\Delta}+\frac{ \Delta_{42}\gamma_{2}}{\Delta}-\frac{2i \Delta_{22}\gamma_{3}}{\Delta}+\frac{2i \Delta_{52}\gamma_{4}}{\Delta}-\frac{2 n\Delta_{32}\gamma_{5}}{\Delta}-\frac{2n \Delta_{62}\gamma_{6}}{\Delta}$$

where

, ,

, ,

, ,

and

, ,

,

,

.

The quantities utilized in Eqs. (41-46) are listed below.

$\Delta=\left|\begin{matrix} \alpha_{11} & \alpha_{12} & \alpha_{13} & 0 & 0 & 0 \\ 0 & 0 & 0 & \alpha_{21} & \alpha_{22} & \alpha_{23} \\ \alpha_{31} & \alpha_{32} & 0 & \alpha_{33} & \alpha_{34} & 0 \\ \alpha_{41} & \alpha_{42} & \alpha_{43} & \alpha_{44} & \alpha_{45} & \alpha_{46} \\ \alpha_{51} & \alpha_{52} & \alpha_{53} & \alpha_{54} & \alpha_{55} & \alpha_{56} \\ \alpha_{61} & \alpha_{62} & \alpha_{63} & \alpha_{64} & \alpha_{65} & \alpha_{66} \end{matrix}\right|$, $\Delta_{11}=\left|\begin{matrix} 0 & 0 & \alpha_{21} & \alpha_{22} & \alpha_{23} \\ \alpha_{32} & 0 & \alpha_{33} & \alpha_{34} & 0 \\ \alpha_{42} & \alpha_{43} & \alpha_{44} & \alpha_{45} & \alpha_{46} \\ \alpha_{52} & \alpha_{53} & \alpha_{54} & \alpha_{55} & \alpha_{56} \\ \alpha_{62} & \alpha_{63} & \alpha_{64} & \alpha_{65} & \alpha_{66} \end{matrix}\right|$, $\Delta_{12}=\left|\begin{matrix} \alpha_{12} & \alpha_{13} & 0 & 0 & 0 \\ \alpha_{32} & 0 & \alpha_{33} & \alpha_{34} & 0 \\ \alpha_{42} & \alpha_{43} & \alpha_{44} & \alpha_{45} & \alpha_{46} \\ \alpha_{52} & \alpha_{53} & \alpha_{54} & \alpha_{55} & \alpha_{56} \\ \alpha_{62} & \alpha_{63} & \alpha_{64} & \alpha_{65} & \alpha_{66} \end{matrix}\right|$, $\Delta_{21}=\left|\begin{matrix} 0 & 0 & \alpha_{21} & \alpha_{22} & \alpha_{23} \\ \alpha_{31} & 0 & \alpha_{33} & \alpha_{34} & 0 \\ \alpha_{41} & \alpha_{43} & \alpha_{44} & \alpha_{45} & \alpha_{46} \\ \alpha_{51} & \alpha_{53} & \alpha_{54} & \alpha_{55} & \alpha_{56} \\ \alpha_{61} & \alpha_{63} & \alpha_{64} & \alpha_{65} & \alpha_{66} \end{matrix}\right|$,$\Delta_{22}=\left|\begin{matrix} \alpha_{11} & \alpha_{13} & 0 & 0 & 0 \\ \alpha_{31} & 0 & \alpha_{33} & \alpha_{34} & 0 \\ \alpha_{41} & \alpha_{43} & \alpha_{44} & \alpha_{45} & \alpha_{46} \\ \alpha_{51} & \alpha_{53} & \alpha_{54} & \alpha_{55} & \alpha_{56} \\ \alpha_{61} & \alpha_{63} & \alpha_{64} & \alpha_{65} & \alpha_{66} \end{matrix}\right|$, $\Delta_{31}=\left|\begin{matrix} 0 & 0 & \alpha_{21} & \alpha_{22} & \alpha_{23} \\ \alpha_{31} & \alpha_{32} & \alpha_{33} & \alpha_{34} & 0 \\ \alpha_{41} & \alpha_{42} & \alpha_{44} & \alpha_{45} & \alpha_{46} \\ \alpha_{51} & \alpha_{52} & \alpha_{54} & \alpha_{55} & \alpha_{56} \\ \alpha_{61} & \alpha_{62} & \alpha_{64} & \alpha_{65} & \alpha_{66} \end{matrix}\right|$, $\Delta_{32}=\left|\begin{matrix} \alpha_{11} & \alpha_{12} & 0 & 0 & 0 \\ \alpha_{31} & \alpha_{32} & \alpha_{33} & \alpha_{34} & 0 \\ \alpha_{41} & \alpha_{42} & \alpha_{44} & \alpha_{45} & \alpha_{46} \\ \alpha_{51} & \alpha_{52} & \alpha_{54} & \alpha_{55} & \alpha_{56} \\ \alpha_{61} & \alpha_{62} & \alpha_{64} & \alpha_{65} & \alpha_{66} \end{matrix}\right|$, $\Delta_{41}=\left|\begin{matrix} 0 & 0 & 0 & \alpha_{22} & \alpha_{23} \\ \alpha_{31} & \alpha_{32} & 0 & \alpha_{34} & 0 \\ \alpha_{41} & \alpha_{42} & \alpha_{43} & \alpha_{45} & \alpha_{46} \\ \alpha_{51} & \alpha_{52} & \alpha_{53} & \alpha_{55} & \alpha_{56} \\ \alpha_{61} & \alpha_{62} & \alpha_{63} & \alpha_{65} & \alpha_{66} \end{matrix}\right|$,$\Delta_{42}=\left|\begin{matrix} \alpha_{11} & \alpha_{12} & \alpha_{13} & 0 & 0 \\ \alpha_{31} & \alpha_{32} & 0 & \alpha_{34} & 0 \\ \alpha_{41} & \alpha_{42} & \alpha_{43} & \alpha_{45} & \alpha_{46} \\ \alpha_{51} & \alpha_{52} & \alpha_{53} & \alpha_{55} & \alpha_{56} \\ \alpha_{61} & \alpha_{62} & \alpha_{63} & \alpha_{65} & \alpha_{66} \end{matrix}\right|$, $\Delta_{51}=\left|\begin{matrix} 0 & 0 & 0 & \alpha_{21} & \alpha_{23} \\ \alpha_{31} & \alpha_{32} & 0 & \alpha_{33} & 0 \\ \alpha_{41} & \alpha_{42} & \alpha_{43} & \alpha_{44} & \alpha_{46} \\ \alpha_{51} & \alpha_{52} & \alpha_{53} & \alpha_{54} & \alpha_{56} \\ \alpha_{61} & \alpha_{62} & \alpha_{63} & \alpha_{64} & \alpha_{66} \end{matrix}\right|$,$\Delta_{52}=\left|\begin{matrix} \alpha_{11} & \alpha_{12} & \alpha_{13} & 0 & 0 \\ \alpha_{31} & \alpha_{32} & 0 & \alpha_{33} & 0 \\ \alpha_{41} & \alpha_{42} & \alpha_{43} & \alpha_{44} & \alpha_{46} \\ \alpha_{51} & \alpha_{52} & \alpha_{53} & \alpha_{54} & \alpha_{56} \\ \alpha_{61} & \alpha_{62} & \alpha_{63} & \alpha_{64} & \alpha_{66} \end{matrix}\right|$, $\Delta_{61}=\left|\begin{matrix} 0 & 0 & 0 & \alpha_{21} & \alpha_{22} \\ \alpha_{31} & \alpha_{32} & 0 & \alpha_{33} & \alpha_{34} \\ \alpha_{41} & \alpha_{42} & \alpha_{43} & \alpha_{44} & \alpha_{45} \\ \alpha_{51} & \alpha_{52} & \alpha_{53} & \alpha_{54} & \alpha_{55} \\ \alpha_{61} & \alpha_{62} & \alpha_{63} & \alpha_{64} & \alpha_{65} \end{matrix}\right|$,$\Delta_{61}=\left|\begin{matrix} \alpha_{11} & \alpha_{12} & \alpha_{13} & 0 & 0 \\ \alpha_{31} & \alpha_{32} & 0 & \alpha_{33} & \alpha_{34} \\ \alpha_{41} & \alpha_{42} & \alpha_{43} & \alpha_{44} & \alpha_{45} \\ \alpha_{51} & \alpha_{52} & \alpha_{53} & \alpha_{54} & \alpha_{55} \\ \alpha_{61} & \alpha_{62} & \alpha_{63} & \alpha_{64} & \alpha_{65} \end{matrix}\right|$

and
